# Molecular characterization based on tumor microenvironment-related signatures for guiding immunotherapy and therapeutic resistance in lung adenocarcinoma

**DOI:** 10.3389/fphar.2023.1099927

**Published:** 2023-01-16

**Authors:** Yamin Jie, Jianing Wu, Dongxue An, Man Li, Hongjiang He, Duo Wang, Anxin Gu, Mingyan E

**Affiliations:** ^1^ Department of Radiation Oncology, The Fourth Affiliated Hospital of Harbin Medical University, Harbin, China; ^2^ Department of Radiation Oncology, Harbin Medical University Cancer Hospital, Harbin, China; ^3^ Department of Endoscopy, Harbin Medical University Cancer Hospital, Harbin, China; ^4^ Department of Head and Neck Surgery, Harbin Medical University Cancer Hospital, Harbin, China; ^5^ Department of Neurology, The 2nd Affiliated Hospital of Harbin Medical University, Harbin, China

**Keywords:** lung adenocarcinoma, molecular subtypes, immunotherapy, cisplatin, paclitaxel, DNA methylation

## Abstract

**Background:** Although the role of tumor microenvironment in lung adenocarcinoma (LUAD) has been explored in a number of studies, the value of TME-related signatures in immunotherapy has not been comprehensively characterized.

**Materials and Methods:** Consensus clustering was conducted to characterize TME-based molecular subtypes using transcription data of LUAD samples. The biological pathways and immune microenvironment were assessed by CIBERSORT, ESTIMATE, and gene set enrichment analysis. A TME-related risk model was established through the algorithms of least absolute shrinkage and selection operator (Lasso) and stepwise Akaike information criterion (stepAIC).

**Results:** Four TME-based molecular subtypes including C1, C2, C3, and C4 were identified, and they showed distinct overall survival, genomic characteristics, DNA methylation pattern, immune microenvironment, and biological pathways. C1 had the worst prognosis and high tumor proliferation rate. C3 and C4 had higher enrichment of anti-tumor signatures compared to C1 and C2. C4 had evidently low enrichment of epithelial–mesenchymal transition (EMT) signature and tumor proliferation rate. C3 was predicted to be more sensitive to immunotherapy compared with other subtypes. C1 is more sensitive to chemotherapy drugs, including Docetaxel, Vinorelbine and Cisplatin, while C3 is more sensitive to Paclitaxel. A five-gene risk model was constructed, which showed a favorable performance in three independent datasets. Low-risk group showed a longer overall survival, more infiltrated immune cells, and higher response to immunotherapy than high-risk group.

**Conclusion:** This study comprehensively characterized the molecular features of LUAD patients based on TME-related signatures, demonstrating the potential of TME-based signatures in exploring the mechanisms of LUAD development. The TME-related risk model was of clinical value to predict LUAD prognosis and guide immunotherapy.

## Introduction

Lung cancer consists of the largest population of all cancers worldwide, where lung adenocarcinoma (LUAD), as the most common histological type, contributes to a proportion of approximately 40% in all lung cancer cases (Sung et al., 2021). The common risk factors are smoking, the exposure to environmental carcinogens, and genetic susceptibility (Gibelin and Couraud, 2016). A large number of lung cancer patients are diagnosed at a late stage, leading to a low 5-year survival rate no more than 20%. (Senosain and Massion, 2020). In the recent years, molecular profiling of lung cancer promotes the development and improvement of molecular-targeted therapy and immunotherapy (Network, 2014; Saito et al., 2018). A diversity of molecular biomarkers of LUAD have been discovered involving transcriptional alteration, genetic mutations, copy number variations, and epigenetics features (Devarakonda et al., 2015; Daugaard et al., 2016; Calvayrac et al., 2017; Hua et al., 2020).

Molecular biomarkers help to predict the prognosis of cancer patients or even are capable to assist decision-makings in clinical treatment. Advanced or metastatic LUAD patients can benefit little from traditional therapy, while the rising immunotherapy or other targeted therapy maybe can function on these patients. For example, immune checkpoint blockade is a hot therapeutic strategy, such as programmed cell death protein 1/programmed cell death ligand 1 (PD-1/PD-L1) inhibitors exhibiting a favorable performance in cancer immunotherapy (Borghaei et al., 2015; Herbst et al., 2016; Jain et al., 2018). However, resistance or immune escape to immunotherapy is a common issue resulting in its inefficiency and poor outcomes. The feature of tumor microenvironment (TME) is one of the critical factors contributing to different response to immunotherapy (Binnewies et al., 2018). For example, high expression of PD-1/PD-L1 is associated with high efficiency of anti-PD-1/PD-L1 therapy (Brody et al., 2017). Cytokines and chemokines released by immune cells, neoplastic or stromal cells can orchestrate and reconstruct the immune microenvironment, and thus lead to different anti-tumor responses (Nisar et al., 2021). Cytokines such as tumor necrosis factor (TNF)-α (Laha et al., 2021), interleukin (IL) family (Sato et al., 2011; Kitamura et al., 2017), and transforming growth factor (TGF)-β (Yao et al., 2010) play an important role in angiogenesis, immune evasion, resistance to immunotherapy, and epithelial-mesenchymal transition (EMT) process responsible for tumor progression and metastasis. Consequently, TME-related features largely determine the anti-tumor response and the activated response to immunotherapy.

Bagaev et al. collected a total of 29 knowledge-based functional gene expression signatures related to TME from previous studies, and grouped them into four classes including anti-tumor microenvironment (e.g., T cells), pro-tumor microenvironment (e.g., macrophages), angiogenesis fibrosis (e.g., angiogenesis), and malignant cell properties (e.g., EMT signature) (Bagaev et al., 2021). Based on these TME-related signatures, they identified four microenvironment subtypes and comprehensively elucidate the relation between TME and melanoma by using transcriptomic and genomic data. The four microenvironment subtypes were also conserved in other cancer types, and were correlated with the response to immunotherapy. The ASLC/ATS/ERS classification is a significant improvement in the classification criteria for lung adenocarcinoma, encompassing pathology, molecular biology, radiology, oncology and clinical practice to provide better clinical diagnosis (Yoshizawa et al., 2011; Gu et al., 2013). The current molecular classification still has limitations on the prognosis evaluation of lung adenocarcinoma patients, such as, the new classification content is too complex to apply. Inspiring by the above study, we sought to explore the TME in LUAD through analyzing these TME-related signatures, and identify effective prognostic genes for guiding immunotherapy or other therapy in LUAD patients.

## Materials and Methods

### Data collection and data preprocessing

TCGA-LUAD dataset (abbreviated as TCGA dataset in the following) containing the RNA sequencing (RNA-seq) data and clinical information of LUAD samples was obtained from The Cancer Genome Atlas (TCGA) database through Sangerbox platform (http://vip.sangerbox.com/) (Shen et al., 2022). mRNA expression was quantified with fragments per kilobase of exon per million reads mapped (FPKM), which converted into transcripts per million (TPM). GSE72094 (Schabath et al., 2016) and GSE50081 (Der et al., 2014) datasets containing microarray data were obtained from Gene Expression Omnibus (GEO) database. In TCGA dataset, LUAD samples with survival status and survival time were included. The average expression value was used when one gene had multiple ensemble IDs. For microarray data, probes were annotated by the annotation profile of corresponding chip platform. If a gene had multiple probes, the averaged value was used. After data preprocessing, 487 LUAD samples were remained in TCGA dataset, 442 and 127 LUAD samples were remained in GSE72094 and GSE50081 datasets respectively.

#### Identification of TME-based molecular subtypes

A total of 29 TME signatures were obtained from the previous research (Bagaev et al., 2021), including four groups of signatures, anti-tumor microenvironment (MHCⅠ, MHCⅡ, coactivation molecules, cytotoxic cells, T cells, T cells trafficking, B cells, M1 signature, NK cells, Th1 signature, and anti-tumor cytokines), pro-tumor microenvironment (Treg, Treg traffic, MDSC, MDSC traffic, neutrophil signature, granulocyte traffic, macrophages, Th2 signature, macrophages/DC traffic, and pro-tumor cytokines), angiogenesis fibrosis (angiogenesis, endothelium, cancer-associated fibroblasts (CAFs), matrix, matrix remodeling), and malignant properties (proliferation rate signature and EMT).

The enrichment score of 29 TME signatures was measured by single sample gene set enrichment analysis (ssGSEA) (Hänzelmann et al., 2013). ConsensusClusterPlus R package (Wilkerson and Hayes, 2010) was utilized to construct consensus matrix based on the ssGSEA score of TME signatures in TCGA dataset. 1 - Pearson correlation was selected as distance and KM algorithm was used for repeating 500 times of bootstraps with each bootstrap having 80% samples of TCGA dataset. The optimal cluster number (k) was selected according to the cumulative distribution function (CDF) curves and consensus matrix.

For the validation of the TME-based subtyping in the external datasets (GSE72094 and GSE50081), a support vector machine (SVM) model was used (Huang et al., 2018) (LUAD samples within TCGA dataset were randomly grouped into training set and testing set with a ratio of 7: 3).

### Functional enrichment analysis

Gene sets of Kyoto Encyclopedia of Genes and Genomes (KEGG) pathways were obtained from Molecular Signature Database (MSigDB), and used for GSEA by “fgsea” algorithm (Liberzon et al., 2015). GSVA R package (Hänzelmann et al., 2013) was utilized to conduct ssGSEA on hallmark pathways obtained from MSigDB and 11 oncogenic pathways (EGFR, hypoxia, NFκB, PI3K, JAK-STAT, MAPK, TGF-β, Trail, VEGF, TNF-α, and P53) obtained from the previous research (Schubert et al., 2018).

#### DNA methylation analysis

In order to observe the methylation difference of different subtypes, we obtained the methylation data set of HumanMethylation450 from the TCGA database, extracted the methylation signals of each sample, and completed the missing values using the KNN method. Limma was used to analyze the methylation difference of each subtype (P.val< 0.05 and |logFC|>log2 (1.1)). In addition, we annotated the methylation sites to the gene promoter region to obtain genes regulated by methylation, The Biological Pathway to Obtain Methylation Disorder of Each Subtype by Function Enrichment Analysis.

### Prediction of the response to immunotherapy and chemotherapeutic drugs

The gene signatures of T cell inflamed GEP (Ayers et al., 2017), Th1/IFN-γ (Danilova et al., 2019), and cytolytic activity (Rooney et al., 2015) were obtained from previous studies. SsGSEA was conducted on these gene signatures. The estimated sensitivity of different groups to chemotherapeutic drugs was evaluated by pRRophetic R package (Geeleher et al., 2014). TIDE algorithm (Jiang et al., 2018) was implemented to analyze immunosuppressive cells and T cell status for estimating immune escape to immunotherapy. Higher TIDE score represents higher immune escape. The immune infiltration and stromal infiltration were evaluated by ESTIMTAE analysis (Yoshihara et al., 2013). CIBERSORT algorithm (Chen et al., 2018) was performed to analyze the proportion of 22 immune-related cells. IMvigor210 dataset (Balar et al., 2017) (treated by anti-PD-L1 therapy) was included to assess the effectiveness of the risk model in predicting prognosis and response to immunotherapy. Drug sensitivity data was downloaded from Genomics of Drug Sensitivity in Cancer (GDSC) database (https://www.cancerrxgene.org/) (Yang et al., 2013).

### Construction and validation of a TME-based risk model

Firstly, differential analysis was performed between different subtypes through limma R package (Ritchie et al., 2015), and differentially expressed genes (DEGs) were screened under criterions of |log2 (fold change, FC)| > 1 and false discovery rate (FDR) < .05. Functional analysis of DEGs including the enrichment analysis of Gene Ontology (GO) terms and KEGG pathways was carried out by ClusterProfiler R package (Yu et al., 2012). The DEGs significantly associated with prognosis (*p* < 0.05) was screened by univariate Cox regression analysis. Least absolute shrinkage and selection operator (Lasso) regression analysis (Friedman et al., 2010) and stepwise Akaike information criterion (stepAIC) (Zhang, 2016) were conducted to compress the number of prognostic genes. The formula of risk model was defined as: risk score = *Σ*(βi*expi), where i represents genes, β represents Lasso coefficients, and exp represents gene expression levels.

TCGA dataset was set as the training set. GSE72094 and GSE50081 datasets were set as the validation set. Each sample obtained a risk score and the risk score was transferred to z-score. The samples were stratified into high-risk (z-score > 0) and low-risk (z-score <0) groups. The effectiveness and efficiency of the risk model was validated by Kaplan-Meier survival analysis and receiver operation characteristic (ROC) curve analysis.

### Statistical analysis

Statistical analysis in this study was conducted in R software (v4.2.0). Wilcoxon test was employed to detect the difference between two groups. The difference among multiple groups was examined by Kruskal–Wallis test. Log-rank test was conducted in survival analysis. We determined *p* < 0.05 as statistically significant.

## Results

### TME signatures were associated with LUAD prognosis

**FIGURE 1:**
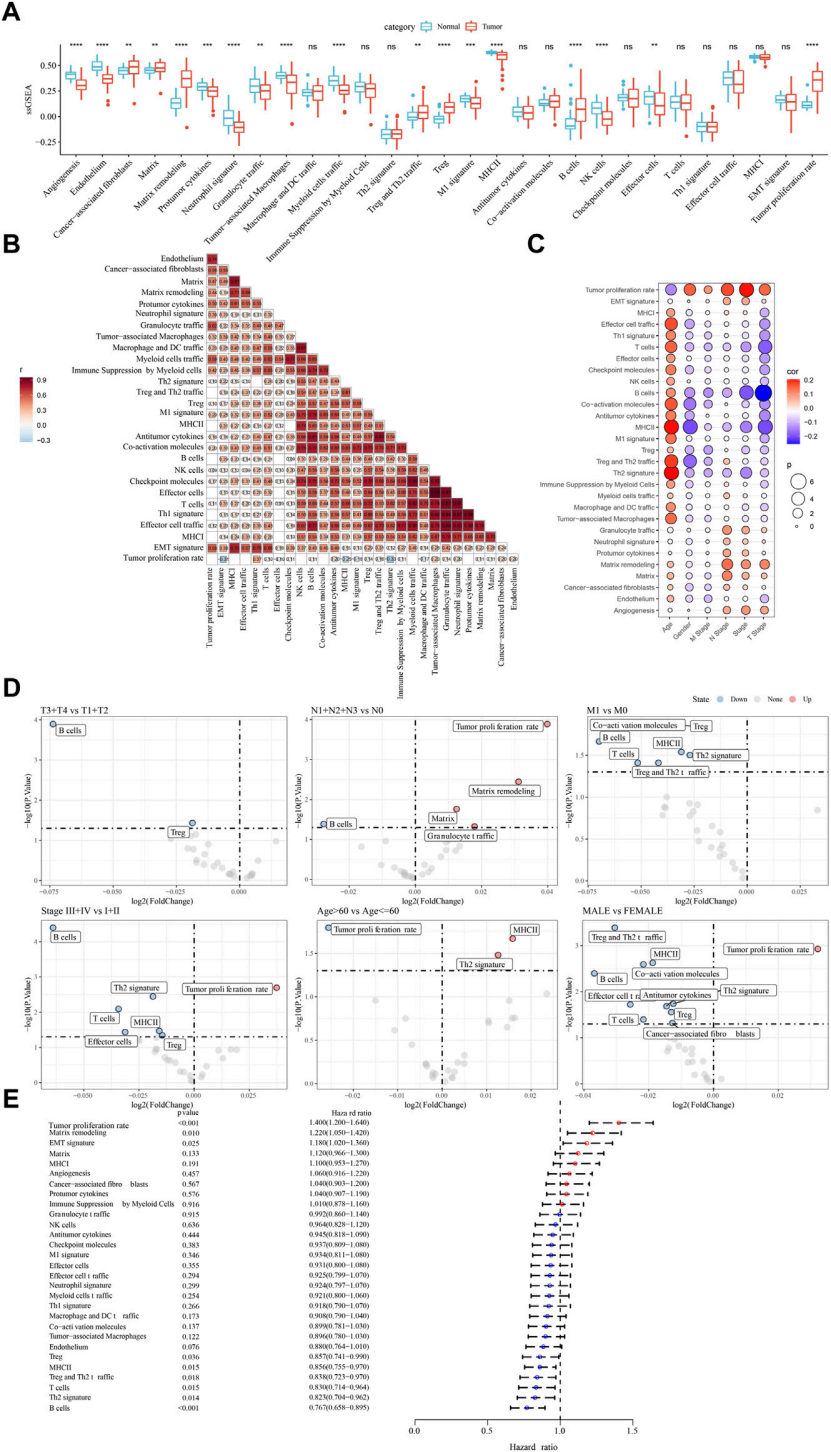
The relation between TME signatures and LUAD analyzed in TCGA dataset **(A)** The ssGSEA score of TME signatures in tumor and normal samples **(B)** Correlation analysis among TME signatures **(C)** Correlation between TME signatures and clinical characteristics **(D)** Fold change of the enrichment of TME signatures in different stages, ages, and genders **(E)** Hazard ratio of 29 TME signatures. ns, not significant. **p < 0.01, ***p < 0.001, ****p < 0.0001.

We compared the enrichment of 29 TME signatures in normal and LUAD samples using ssGSEA. 18 TME signatures were significantly different in normal and tumor samples (Figure 1A). Stromal-related signatures such as CAFs (*p* < 0.01), matrix (*p* < 0.01), and matrix remodeling (*p* < 0.0001) were more enriched in tumor samples compared to the normal. In addition, pro-tumor signatures such as regulatory T cells (Tregs) and malignant cell properties such as tumor proliferation rate were more accumulated in tumor samples (*p* < 0.0001). Correlation analysis on these 29 TME signatures revealed evidently positive correlations among them, suggesting close interactions among these signatures (Figure 1B). We assessed the relation between the signatures and clinical characteristics, and found that MHC Ⅱ and Th2 signature were positively correlated with age (Figures 1C,D). Anti-tumor signatures such as T cells, B cells, coactivation molecules, and MHC Ⅱ were negatively correlated with gender, T stage, N stage, M stage, and Stage. Notably, tumor proliferation rate was significantly upregulated in N1-N3 stages and Stage Ⅲ+Ⅳ (Figure 1D). In the relation of 29 TME signatures to LUAD overall survival, we found that some of them were risk factors such as tumor proliferation rate, matrix remodeling, and EMT signature and some were protective factors such as B cells, Th2 signature, T cells, and MHC Ⅱ (*p* < 0.05, Figure 1E), indicating a close relation between TME signatures and LUAD prognosis.

#### Identification of TME-based molecular subtypes

**FIGURE 2:**
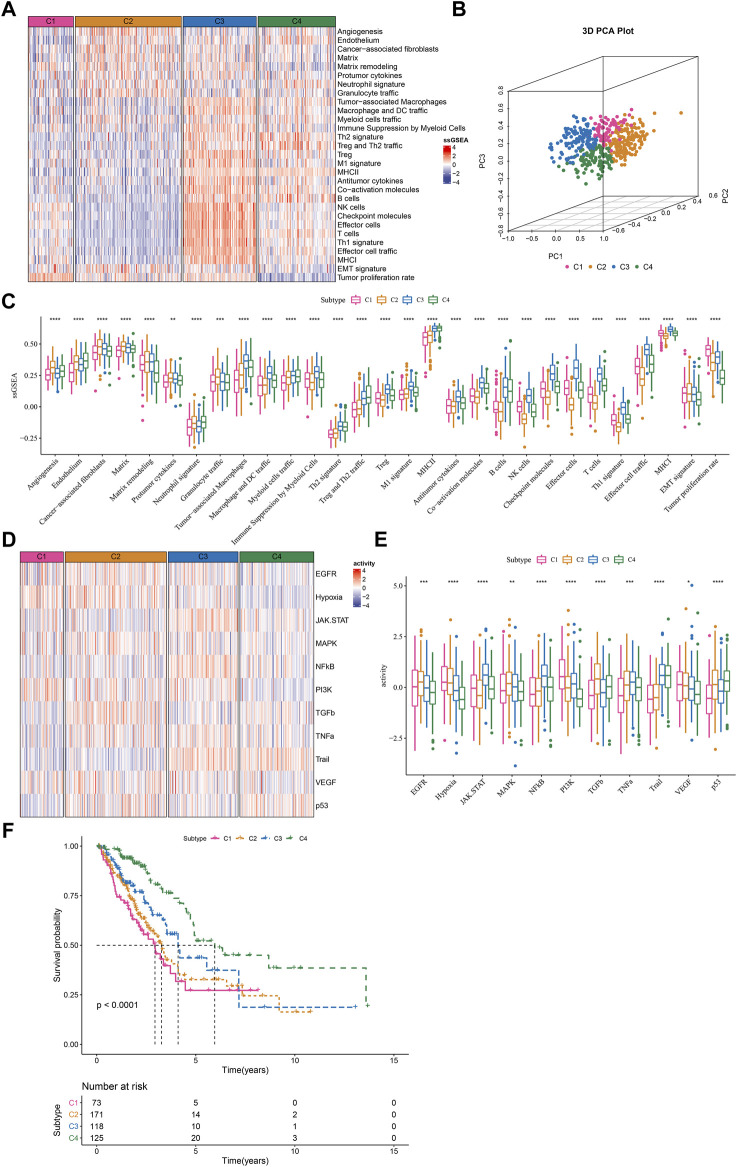
The immune and pathway difference of four TME-based molecular subtypes in TCGA dataset **(A)** The heatmap showed the enrichment of TME signatures in four subtypes **(B)** 3D PCA plot of four subtypes based on TME signatures **(C)** The ssGSEA score of TME signatures in four subtypes **(D)** The heatmap showed the enrichment of oncogenic pathways in four subtypes **(E)** The ssGSEA score of oncogenic pathways in four subtypes **(F)** Kaplan-Meier survival plot of four subtypes. *p < 0.05, **p < 0.01, ***p < 0.001, ****p < 0.0001.

Given that TME signatures were significantly related to LUAD prognosis, we attempted to identify molecular subtypes based on their enrichment scores. By using consensus clustering, we determined four molecular subtypes (C1, C2, C3, and C4) according to CDF and consensus matrix (Supplementary Figure S1). Four subtypes had distinct enrichment patterns of 29 TME signature as shown in the heatmap (Figure 2A). C3 and C4 subtypes had higher enrichment of anti-tumor signatures compared to C1 and C2. C4 subtype had evidently lower enrichment of EMT signature and tumor proliferation rate compared to C1, C2 and C3 subtypes. Principle component analysis (PCA) displayed the different distribution of four subtypes based on the TME signatures (Figure 2B). Significant differences were shown among four subtypes on the enrichment of all TME signatures (Figure 2C). In addition, we analyzed the oncogenic activity of four subtypes, and the result showed different activation of these oncogenic pathways (Figures 2D,E). PI3K and hypoxia were activated in C1, EGFR and TGF-β were activated in C2, JAK-STAT and NFκB were activated in C3, and P53 signaling was activated in C4. Moreover, survival analysis revealed that C1 subtype had the worst prognosis while C4 had the longest overall survival (*p* < 0.0001, Figure 2F). Different activation of these pathways may indicate different TME-based molecular mechanisms of tumor progression. Furthermore, we evaluated the distribution of different clinical characteristics in four subtypes. The result exhibited an evident trend that the samples with advanced stages were more distributed in C1 (Supplementary Figure S2). Female patients and the patients with age >60 had a higher proportion in C4 compared with that in C1-C3 (Supplementary Figure S2). Not surprisingly, C1 had the largest number of the samples with dead status than other subtypes (Supplementary Figure S1).

#### Genomic landscape and DNA methylation of four TME-based subtypes

**FIGURE 3:**
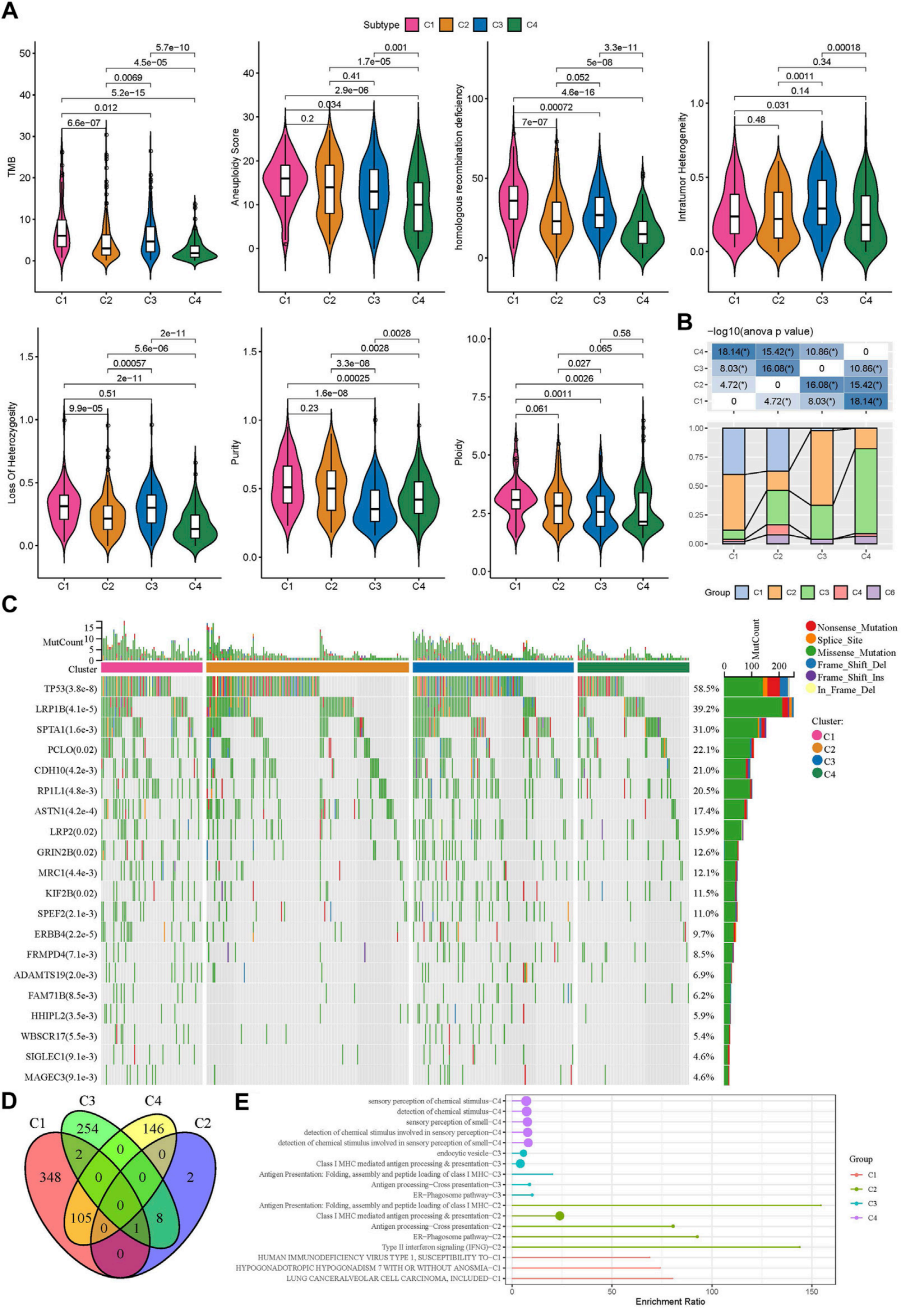
Gene mutations and genomic features of four subtypes in TCGA dataset **(A)** The score of TMB, aneuploidy, homologous recombination deficiency, intratumor heterogeneity, loss of heterozygosity, tumor purity, and ploidy in four subtypes **(B)** The distribution of previously reported immune subtypes (C1, C2, C3, C4, and C6) in TME-based subtypes **(C)** The top 20 mostly mutated genes in LUAD **(D)** Cross Venn Diagram of Different Methylation Sites of Each Subtype **(E)** Biological pathway of significant enrichment of methylation sites in different subtypes (top5).

Genomic instability has been demonstrated to be associated with tumor development. We obtained a series of genomic characteristics of TCGA-LUAD data from a pan-cancer research (pan-cancer) (Thorsson et al., 2018). C1 subtype had relatively high scores of tumor mutation burden (TMB), aneuploidy, homologous recombination deficiency, loss of heterozygosity, purity, and ploidy, while C3 showed relatively high score of intratumor heterozygosity (Figure 3A). In the previous pan-cancer research, they identified six immune subtypes of LUAD (C1, C2, C3, C4, and C6 immune subtypes). We analyzed the distribution of previous immune subtypes in our TME-based subtypes (Figure 3B). C1 immune subtype (also known as wound healing) mostly distributed in C1 and C2 TME-based subtypes. C2 immune subtype (also known as IFN-γ dominant) mostly accumulated in C1 and C3 TME-based subtypes. C4 TME-based subtype had the highest proportion of C3 immune subtype (also known as inflammatory). The different distribution of previous immune subtypes in our TME-based subtypes also supported the distinct TME characteristics of four subtypes. In addition, we evaluated the gene mutations in four subtypes, and observed that TP53, LRP1B, and SPTA1 contributed high somatic mutation frequencies (Figure 3C). In addition, we analyzed the different DNA methylation sites of each subtype in the genome. Among them, C1 has the most differential methylation sites, C2 has only a small amount of DNA methylation differences, and the different methylation sites of each subtype overlap less (Figure 3D). Further functional analysis showed that the methylation site of C1 imbalance was mainly related to LUNG CANCERALVEOLAR CELL CARCINOMA, INCLUDED, the methylation site of C2 imbalance was mainly related to Type II interaction signaling (IFNG), the methylation site of C3 imbalance was mainly related to ER Phagosome pathway, and the methylation site of C4 imbalance was mainly related to detection of chemical stimulus involved in sensor performance of smart (Figure 3E), These results indicate that different molecular subtypes may have different apparent disorder patterns.

#### Four TME-based subtypes had differently activated pathways

**FIGURE 4:**
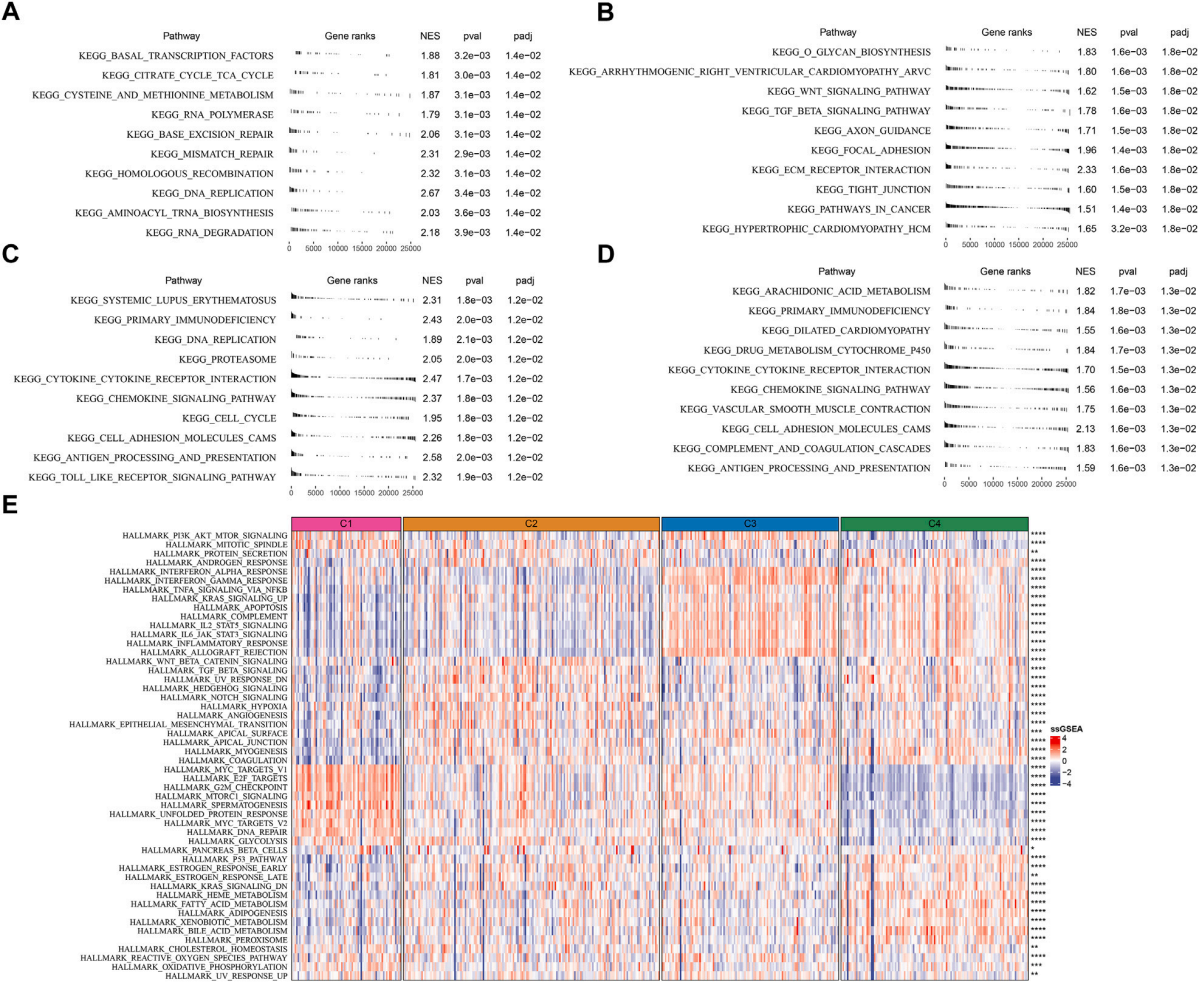
Analysis of KEGG and hallmark pathways in TCGA dataset **(A–D)** GSEA revealed the top 10 enriched KEGG pathways of C1 **(A)**, C2 **(B)**, C3 **(C)**, and C4 **(D, E)** The heatmap showed the enrichment of hallmark pathways. Comparison of the enrichment score among four subtypes was performed and the significance was shown in the right. *p < 0.05, **p < 0.01, ***p < 0.001, ****p < 0.0001.

To further understand the different molecular mechanism of tumor development in four subtypes, we analyzed the biological pathways using GSEA. Different pathways were enriched in four subtypes. In C1 subtype, cell cycle and DNA repair-related pathways were relatively activated such as mismatch repair, base excision repair, homologous recombination, and DNA replication (Figure 4A). In C2 subtype, EMT-related pathways were significantly enriched such as ECM receptor interaction, tight junction, TGF-β signaling pathway, and focal adhesion (Figure 4B). In C3 subtype, cell cycle and immune-related pathways were activated such as DNA replication, cell cycle, cytokine-cytokine receptor interaction, antigen processing and presentation, chemokine signaling pathway, and Toll-like receptor signaling pathway (Figure 4C). In C4 subtype, immune-related pathways were also evidently activated such as chemokine signaling pathway, antigen processing and presentation, cytokine-cytokine receptor interaction, and complement and coagulation cascades (Figure 4D).

Additionally, similar results were carried out in hallmark pathways (Figure 4E). Cell cycle-related pathways were much enriched in C1 subtype. C2 subtype showed activated EMT, angiogenesis, hypoxia, Notch signaling, and TGF-β signaling. Immune-related pathways were significantly enriched in both C3 and C4 subtypes. Besides, C4 subtype also had relatively high enrichment of metabolic pathways such as heme metabolism, fatty acid metabolism, adipogenesis, xenobiotic metabolism, and bile acid metabolism. The above results implied that these differently enriched pathways may result in different TME characteristics in four subtypes.

### Different response of four TME-based subtypes to immunotherapy and chemotherapeutic drugs

**FIGURE 5:**
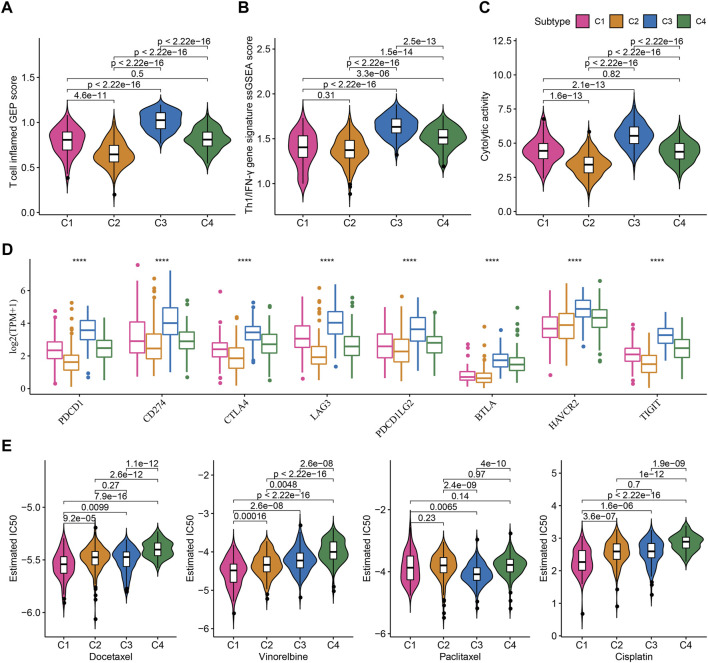
Prediction of the response to immunotherapy and chemotherapeutic drugs in TCGA dataset **(A–C)** The ssGSEA score of T cell inflamed GEP, Th1/IFN-γ, and cytolytic activity in four subtypes **(D)** The expression of immune checkpoint genes in four subtypes **(E)** The estimated IC50 of four chemotherapeutic drugs in four subtypes. ****p < 0.0001.

TME characteristics can decide the outcomes of clinical treatment to some extent especially immunotherapy. We selected three immune-related signatures including T cell inflamed gene expression profiles (GEP), Th1/IFN-γ, and cytolytic activity from previous studies to evaluate the predicted response to immunotherapy. T cell inflamed GEP has been illustrated to reflect the response to immune checkpoint inhibitors (ICIs) (Ott et al., 2019). IFN-γ is an important cytokine in modulating immune response and anti-tumor activity (Danilova et al., 2019). Cytolytic activity reflects the cytotoxicity of activated T cells (Rooney et al., 2015). The above three signatures manifested differences in four subtypes, with that C3 subtype had the highest ssGSEA score of T cell inflamed GEP, IFN-γ, and cytolytic activity (Figures 5A–C). Immune checkpoints are also important in the response to ICIs. High expression of PD-1/PD-L1 indicates high response to ICIs. Analysis on immune checkpoints clarified that C3 subtype had the highest expression levels of PDCD1 (PD-1), CD274 (PD-L1), CTLA4, LAG3, PDCD1LG2, BTLA, HAVCR2, and TIGIT (*p* < 0.0001, Figure 5D), meaning that C3 subtype was predicted to have the highest sensitivity to immune checkpoint blockade treatment. Furthermore, we examined the estimated IC50 of four chemotherapeutic drugs including Docetaxel, Vinorelbine, Paclitaxel, and Cisplatin. C1 subtype had the lowest estimated IC50 of Docetaxel, Vinorelbine, and Cisplatin, and C3 subtype had the lowest IC50 of Paclitaxel (*p* < 0.01, Figure 5E). The results suggested that C1 may benefit much from the treatment of Docetaxel, Vinorelbine, and Cisplatin, and C3 may benefit much from Paclitaxel.

### Validation the robustness of TME-based subtypes in two external datasets

**FIGURE 6:**
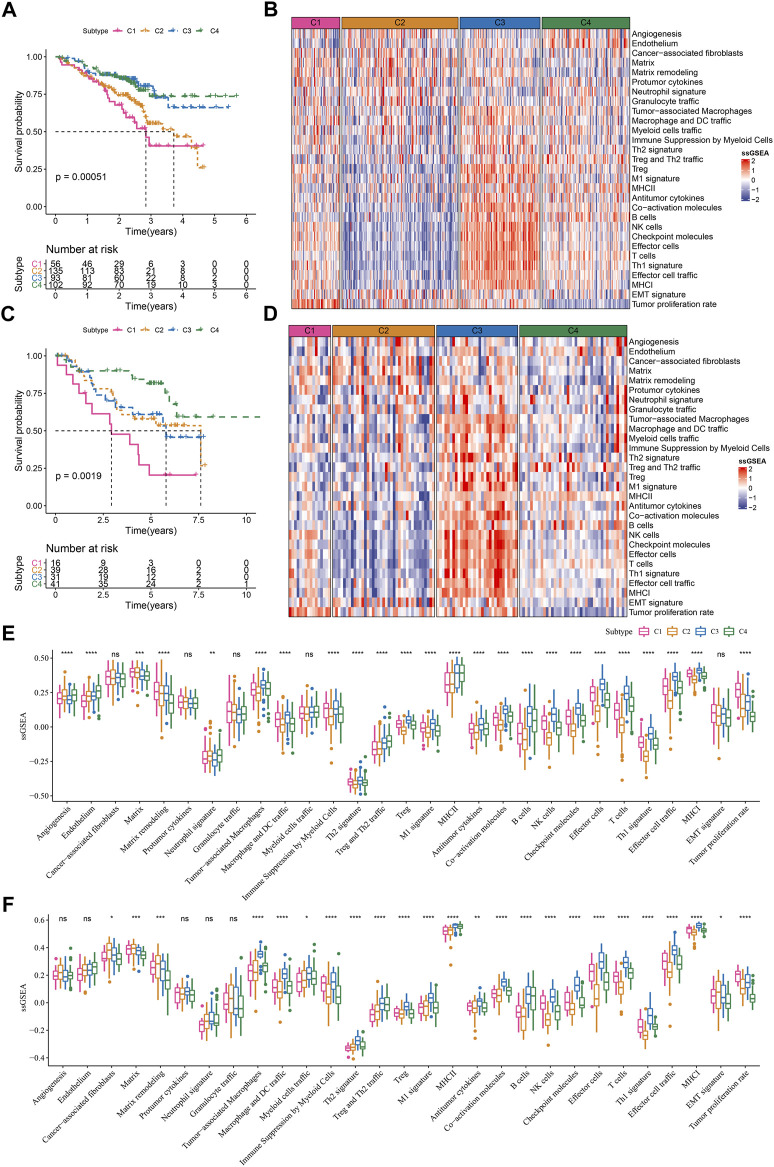
Validation of TME-based molecular subtypes in two external datasets **(A)** Survival plot of four subtypes in GSE72094 dataset **(B)** The heatmap showed the enrichment of TME signatures in GSE72094 dataset **(C)** Survival plot of four subtypes in GSE50081 dataset **(D)** The heatmap showed the enrichment of TME signatures in GSE50081 dataset **(E–F)** The ssGSEA score of 29 TME signatures in four subtypes in GSE72094 and GSE50081 datasets. ns, not significant. *p < .05, **p < 0.01, ***p < 0.001, ****p < 0.0001.

LUAD samples in the TCGA dataset were randomly grouped into training cohort (n = 343) and test cohort (n = 144). TME score was inputted to SVM model for determining TME-based subtypes in the training cohort. The accuracy of the SVM model in the test cohort was 1. We then used the SVM model to examine the TME-based subtypes in the external datasets (GSE72094 and GSE50081). The external datasets showed the similar results on the TME score of four subtypes compared to the result in the TCGA dataset (Figures 6A–D). C1 subtype showed the worst prognosis and C4 subtype had the best prognosis in both two external datasets (Figures 6A,C). The enrichment patterns of 29 TME signatures in GSE72094 and GSE50081 datasets were similar to that in TCGA dataset (Figures 6B,D; Figure 2A). Tumor proliferation rate was highly enriched in C1 subtype. Anti-tumor and pro-tumor signatures were both more enriched in C3 compared with other subtypes. The ssGSEA scores of most TME signatures were different among four subtypes in two external datasets (Figures 6E,F). The validation of TME-based subtypes in the external datasets supported the reliability and robustness of the subtyping, and suggested the important role of these TME signatures in LUAD.

#### Development of a TME-based prognostic model

As four TME-based subtypes showed different TME scores, prognosis and activated pathways, we then identified the DEGs among four subtypes. A total of 353 DEGs (135 upregulated and 218 downregulated) were screened in C1 vs other, 91 DEGs (9 upregulated and 82 downregulated) were screened in C2 vs other, 171 DEGs (161 upregulated and 10 downregulated) were screened in C3 vs other, and 396 DEGs (223 upregulated and 173 downregulated) were screened in C4 vs other. Functional analysis on all upregulated DEGs unveiled different biological function of upregulated DEGs in different subtypes. Cell cycle-related pathways and processes were enriched in C1; stromal-related processes were enriched in C2; immune-related pathways and processes were enriched in C3 (Supplementary Figure S3).

**FIGURE 7:**
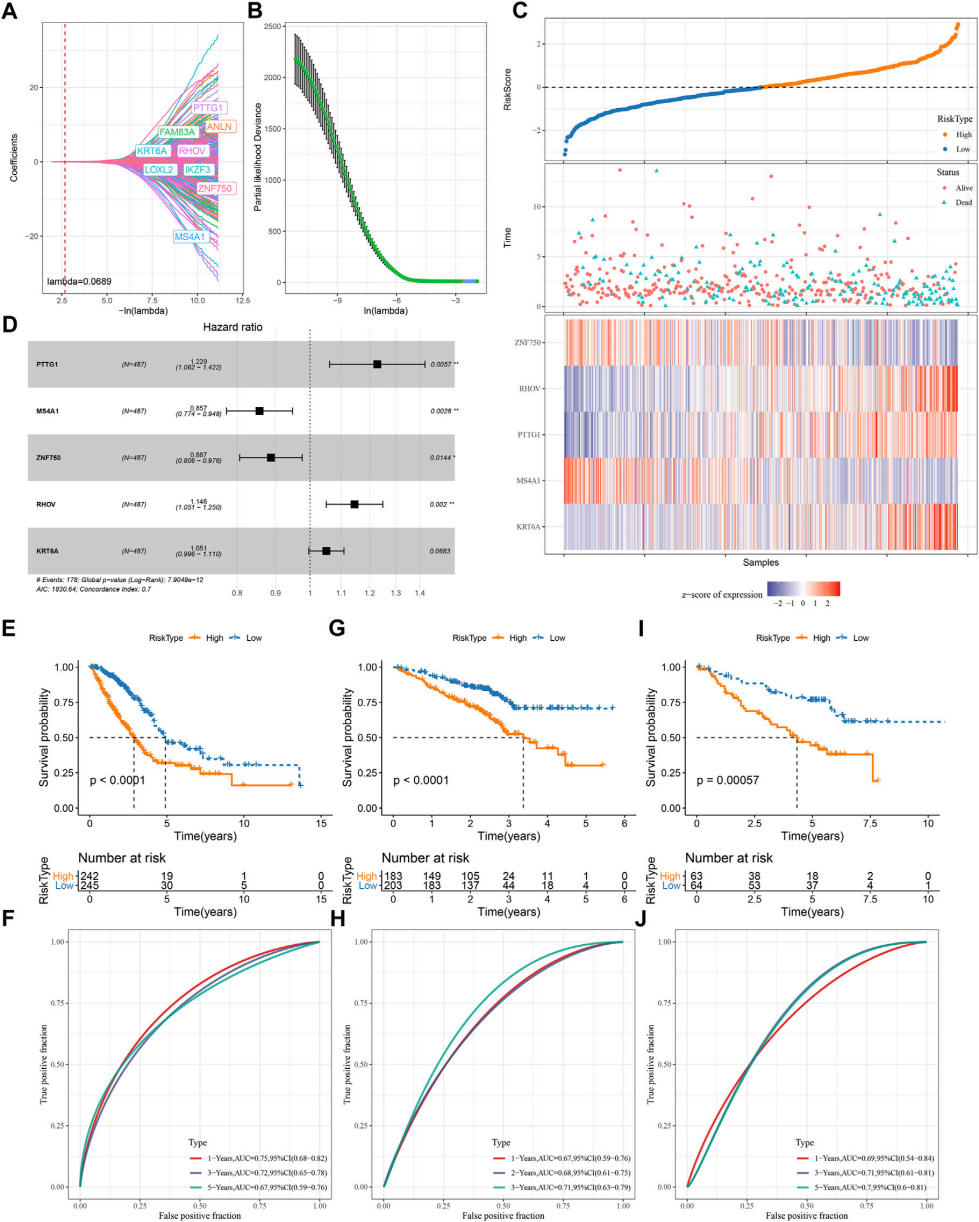
Construction and validation of a TME-based risk model **(A–B)** Lasso regression analysis of prognostic genes. The coefficients changed with increasing lambda value **(A)**. Partial likelihood deviance under different lambda values **(B)**. When lambda = 0.0689 (red dotted line in A and red dot in B), the model reached the optimal **(C)** The forest plot of the final five prognostic genes in the risk model **(D)** The risk score, survival status and expression of five prognostic genes of tumor samples in TCGA dataset **(E–I)** Survival plot of high-risk and low-risk groups in TCGA **(E)**, GSE72094 **(G)**, GSE50081 **(I)** datasets **(F–J)** ROC curve of the risk model in predicting survival in TCGA **(F)**, GSE72094 **(H)**, GSE50081 **(J)** datasets.

We screened a total of 648 DEGs among four subtypes in TCGA dataset after removing the duplicate DEGs. Univariate Cox regression analysis detected a total of 164 risk genes and 254 protective genes within 648 DEGs (*p* < 0.05, Supplementary Table S1). Next, we applied Lasso regression and stepAIC to dig out key prognostic genes from the above risk and protective genes. Lasso analysis compressed the coefficients to zero and remained nine prognostic genes when the lambda value = 0.0689 (Figures 7A,B). Subsequently, stepAIC was performed on the nine prognostic genes and further compress the number of genes. Consequently, five prognostic genes were remained, including PTTG1, MS4A1, ZNF750, RHOV, and KRT6A. The 5-gene prognostic model was determined as: Risk Score = 0.206*PTTG1—0.155*MS4A1—0.12*ZNF750 + 0.136*RHOV + 0.05*KRT6A.

#### Examination the performance of the 5-gene prognostic model

Each LUAD sample obtained a risk score, and was stratified into high-risk and low-risk groups referring z-score = 0 as a cut-off (Figure 7D). Two risk groups exhibited different enrichment pattern of survival status, with a higher density of dead samples in high-risk groups. Five prognostic genes showed distinct expression patterns in two risk groups, where ZNF570 and MS4A1 were relatively upregulated in low-risk group, while RHOV, PTTG1, and KRT6A were upregulated in high-risk group. There were 242 and 245 LUAD samples in high- and low-risk groups respectively, and two groups exhibited distinct prognosis (*p* < 0.0001, Figure 7E). ROC curve analysis revealed that the risk model had favorable AUC in predicting survival at 1, 3, and 5 years with the scores of 0.75, 0.72, and 0.67 respectively (Figure 7F). Furthermore, we validated the 5-gene risk model in two independent datasets (GSE72094 and GSE50081). The validation results were consistent with the TCGA dataset (Figures 7G–J), which demonstrated the effectiveness and reliability of the risk model.

The relation of risk score to different clinical characteristics was assessed and a trend showed that the risk score was higher in the advanced stages compared with early stages (Supplementary Figure S4). A significant difference was also observed in different ages and genders. Moreover, we compared the risk score in four TME-based subtypes, and the results showed that the prognosis of subtypes was consistent with their risk levels. C1 with the worst prognosis showed the highest risk score, which was consistent with the previous results (Supplementary Figure S4; Supplementary Figure S2). In different clinical characteristics, the risk model also showed a favorable performance in dividing samples into high-risk and low-risk groups (Supplementary Figure S4).

#### Biological pathways and immune characteristics of two risk groups

**FIGURE 8:**
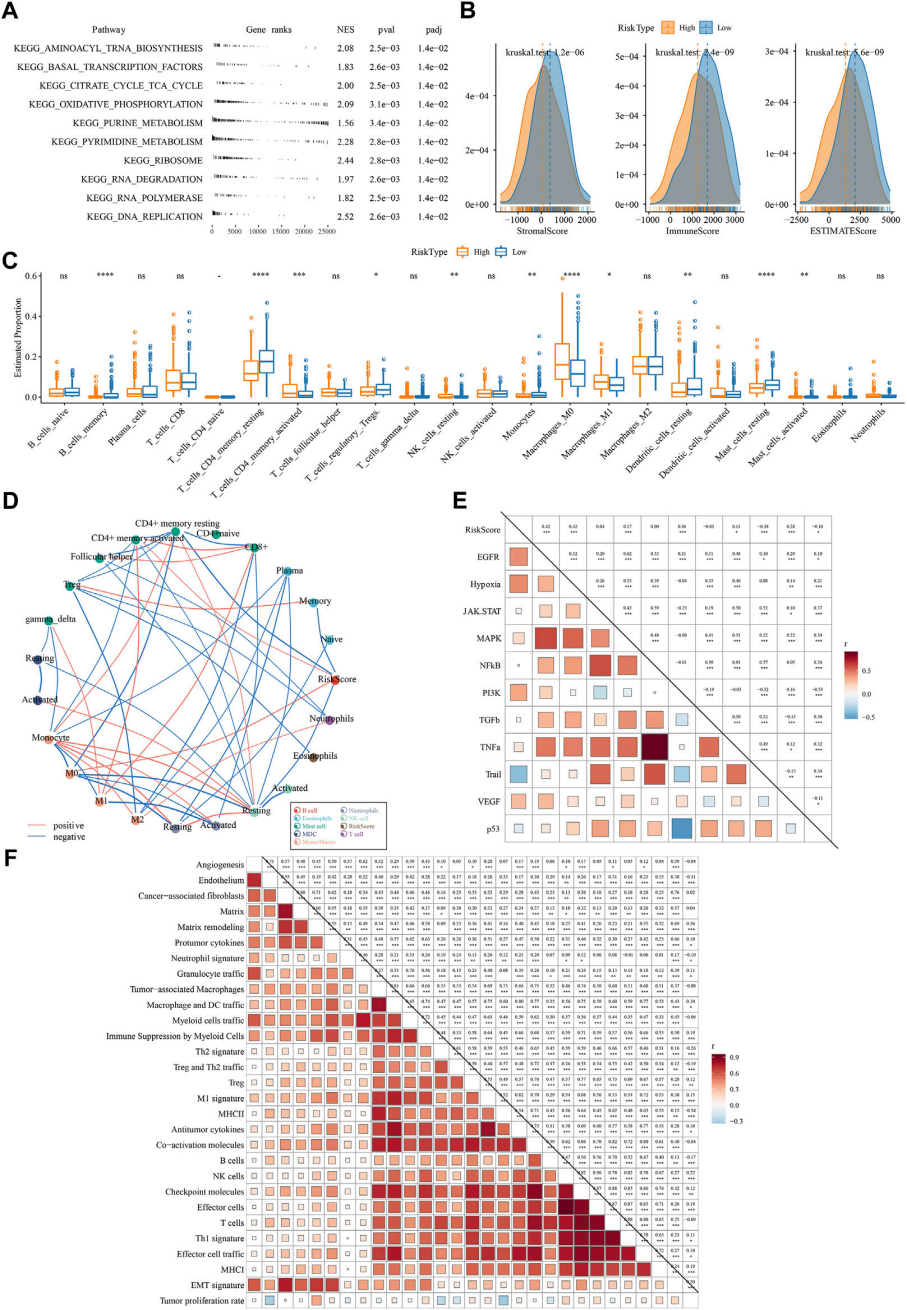
The difference of two risk groups on biological pathways and immune characteristics analyzed in TCGA dataset **(A)** GSEA result showed the significantly enriched KEGG pathways in high-risk group **(B)** The immune score, stromal score and ESTIMATE score calculated by ESTIMATE analysis **(C)** The estimated proportion of 22 immune-related cells analyzed by CIBERSORT **(D)** Correlation analysis among immune cells and risk score. Red and blue lines indicate the positive and negative correlations respectively. The thicker line indicates the stronger correlation **(E)** Correlation analysis among oncogenic pathways and risk score. Red and blue indicate positive and negative correlations respectively. The darker color indicates the stronger correlation. **(F)** the correlation analysis between risk score and 29 TME pathways.

Next, we compared the difference of two risk groups in biological pathways and immune characteristics. GSEA on KEGG pathways revealed that high-risk group had relatively activated pathways of purine metabolism, pyrimidine metabolism, citrate cycle TCA cycle, oxidative phosphorylation, and DNA replication (Figure 8A). Consider that immunity is related to the tumor, thus we determine immune characteristics. In terms of tumor microenvironment, two risk groups had distinguished infiltration levels that low-risk group had higher infiltration of both immune cells and stromal cells than high-risk group (Figure 8B). Of 22 immune cells, 11 immune cells were differentially distributed in two risk groups (Figure 8C). Low-risk group had higher infiltration of resting dendritic cells, memory B cells, resting memory CD4 T cells, and resting mast cells than high-risk group, while M0 macrophages and M1 macrophages were lower enriched in low-risk group. Supportively, risk score was significantly correlated with resting memory CD4 T cells and M0 macrophages (Figure 8D). In the relation of risk score with oncogenic pathways, EGFR, hypoxia, PI3K, and VEGF pathways were positively correlated with risk score (R = 0.42, 0.42, 0.36, and 0.28, respectively) (Figure 8E), suggesting that these pathways may be highly involved in the TME modulation and tumor progression. Moreover, risk score was strongly associated to 29 TME pathways (Figure 8F).

### Different response of two risk groups to immunotherapy and chemotherapeutic drugs

**FIGURE 9:**
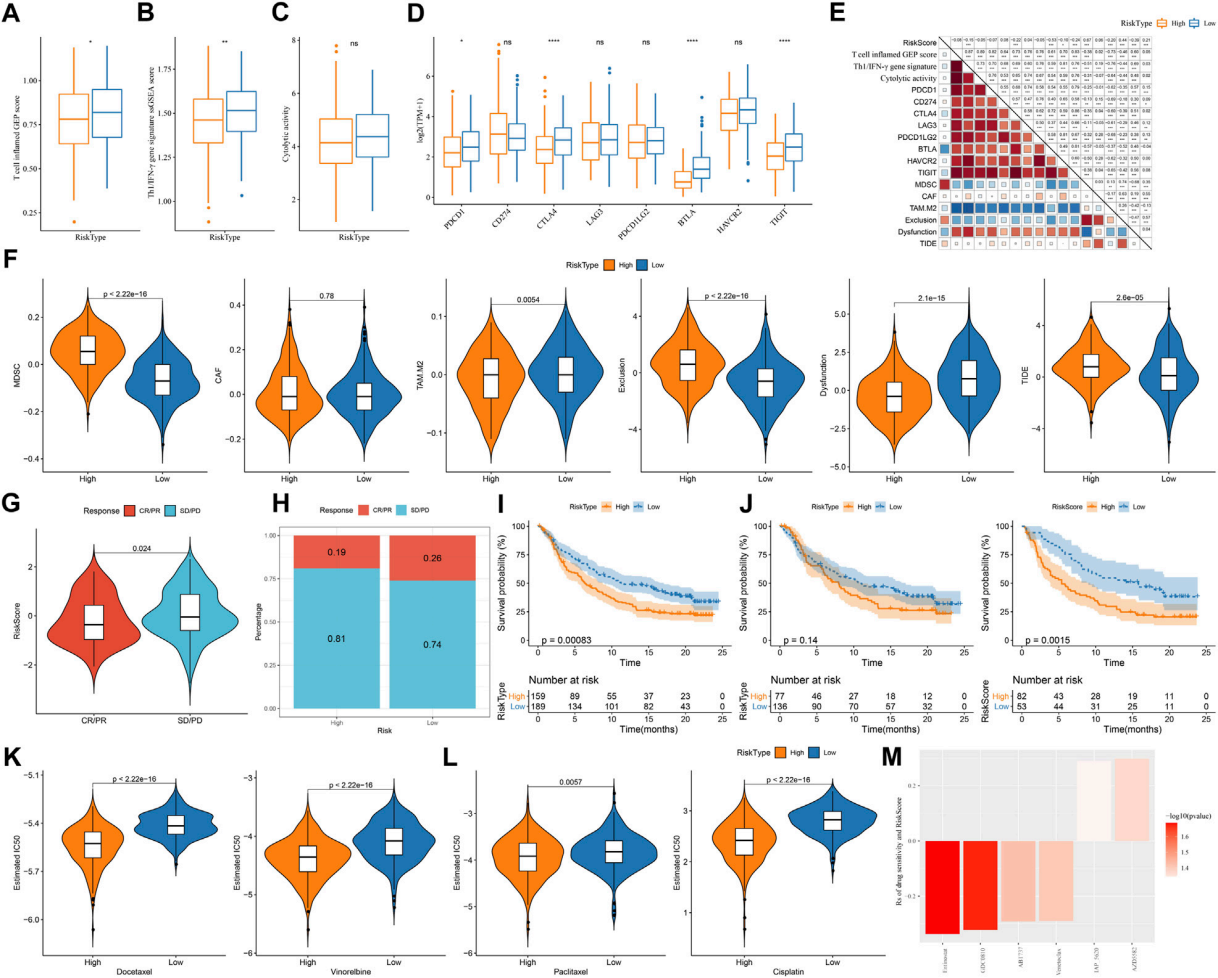
The predicted response to immunotherapy and chemotherapeutic drugs analyzed in TCGA and IMvigor210 datasets **(A–C)** The score of T cell inflamed GEP, Th1/IFN-γ signature, and cytolytic activity in two risk groups **(D)** The expression of immune checkpoint genes in two risk groups **(E)** TIDE analysis calculated the enrichment of immunosuppressive cells, T cell exclusion, T cell dysfunction, and TIDE score in two risk groups **(F)** Correlation of risk score with immunotherapy-related indicators **(G)** The risk score in CR/PR and SD/PD groups in IMvigor210 dataset **(H)** The proportion of CR/PR and SD/PD in two risk groups in IMvigor210 dataset **(I–K)** Survival plot of high-risk and low-risk groups in all stages **(I)**, early stage (I+Ⅱ) **(J)**, and late stage (Ⅲ+Ⅳ) **(K)** in IMvigor210 dataset **(L)** The estimated IC50 of four chemotherapeutic drugs in two risk groups **(M)** Drug sensitivity of potential drugs in the relation to the risk score. Rs < 0 indicates drug sensitivity and Rs > 0 indicates drug resistance.

Assessment on immunotherapy-related indicators unveiled that low-risk group had higher score of T cell inflamed GEP, Th1/IFN-γ, and cytolytic activity (Figures 9A–C), indicating a higher response of low-risk group to immunotherapy than high-risk group. Immune checkpoint analysis showed PD-1, CTLA-4, BTLA, and TIGIT were higher expressed in low-risk group than that in high-risk group (Figure 9D), suggesting that low-risk group was more responsive to immune checkpoint inhibitors. TIDE analysis predicted that high-risk group was more prone to escape from immunotherapy, which may result from its high enrichment of myeloid-derived suppressor cells (MDSCs) and high T cell exclusion (Figure 9E). Correlation analysis of risk score with the above immunotherapy-related indicators showed that risk score was positively correlated with MDSC (R = .67), T cell exclusion (R = .44), and TIDE score (R = .21) but was negatively correlated with BTLA (R = -0.53) and T cell dysfunction (R = -0.39) (Figure 9F). Moreover, risk score was positively correlated to TMB (Supplementary Figure S5), and high group had enhanced TMB (Supplementary Figure S5).

Furthermore, we used an immunotherapy dataset (IMvigor210) to validate the reliability of the risk model in predicting immune response. Risk score was significantly higher in SD/PD group compared with that in CR/PR group (*p* = 0.024, Figure 9G), and low-risk group also had a higher proportion of CR/PR (*p* = 0.2325302, Figure 9H). The risk model was also effective to distinguish high-risk patients receiving immunotherapy in IMvigor210 dataset, especially in the patients with late stages (Figures 9I–K). In addition, we evaluated the ability of the risk model in predicting the response to chemotherapeutic drugs in TCGA dataset. High-risk group had significantly lower estimated IC50 of all four chemotherapeutic drugs (Figure 9L), implying that high-risk group was more sensitive to these four drugs than low-risk group. By utilizing the drug sensitivity data in GDSC database, we identified six drugs significantly correlating with risk score where four drugs (entinostat, GDC0810, ABT737, and venetoclax) may serve as therapeutic drugs for LUAD (Figure 9M).

## Discussion

In the present study, we used 29 TME-related signatures as a basis to identify TME-based molecular subtypes for LUAD. Four TME-based subtypes were identified and their clinical and molecular features such as survival time, gene mutations, genomic characteristics, immune infiltration, and biological pathways were characterized. Four subtypes showed distinct clinical and molecular features, as well as different response to immunotherapy and chemotherapeutic drugs. By comparing the expression profiles between different subtypes, we identified DEGs and screened five key prognostic genes to construct a TME-related risk model for predicting LUAD prognosis.

Among 29 TME-related signatures, tumor proliferation rate, EMT signature, and matrix remodeling were shown to be positively correlated with poor prognosis. EMT has been widely known as a promotive process in inducing tumor cell invasion and metastasis through weakening cell-cell adhesion (Ye and Weinberg, 2015). The junctions of mesenchymal cells with extracellular matrix are loose, which enable tumor cells easily to migrate. Tumor proliferation rate had the highest HR (1.40) among these signatures, in accordant with the close relation between tumor proliferate rate and stage. Evidently positive correlations were observed among 29 TME-related signatures, suggesting a complicated regulation system of TME. Therefore, we used these TME-related signatures as a basis to perform molecular subtyping for LUAD patients.

We identified four TME-based molecular subtypes and each subtype showed different enrichment patterns of TME-related signatures. C1 subtype had the highest enrichment of tumor proliferation rate, which was considered as a pro-tumor phenotype. C2 subtype had the least infiltration of anti-tumor immune cells or molecules, and relatively high enrichment of angiogenesis (Voron et al., 2014), CAFs (Ziani et al., 2018), and pro-tumor cytokines, which was suggested as an immune-suppressed phenotype. C3 subtype had the highest enrichment of anti-tumor cells but the immunosuppressive cells or signatures such as tumor-associated macrophages (Pan et al., 2020), Treg (Tanaka and Sakaguchi, 2017), and checkpoint molecules were also highly enriched. C3 subtype was considered as an immune infiltrated phenotype. C4 subtype had the lowest enrichment of tumor proliferation rate and EMT signature. Therefore, we suggested C4 subtype as a tumor-silent phenotype. Survival analysis of four subtypes showed that C1 had the worst survival and C4 had the longest survival, which was consistent with their TME-related features.

Genomic instability is an important feature and is considered as a hallmark in cancers (Negrini et al., 2010). The mutation of oncogenes promotes DNA damage and genomic arrangements in cancer (Tubbs and Nussenzweig, 2017). High non-synonymous TMB was demonstrated to be associated with favorable prognosis in resected non-small cell lung cancer patients (Devarakonda et al., 2018). In our results, C1 had the highest score of aneuploidy, homologous recombination deficiency, loss of heterozygosity and ploidy, indicating high genomic instability thus contributing to poor prognosis of C1. Although high TMB was also shown in C1, the large number of genomic alterations covered the beneficial effect of TMB. The contribution of TME in genomic instability has been revealed in recent years, and hypoxia is a main factor causing DNA damage and genomic instability (Sonugür and Akbulut, 2019). In pathway analysis, we found that two hypoxia-related pathways, reactive oxygen species pathway and oxidative phosphorylation, were relatively activated in C1, which supported the above observation.

Biological pathway analysis revealed that four TME-based subtypes had different activated pathways that may lead to their different outcomes. In C1 subtype, cell cycle-related pathways such as E2F targets, G2M checkpoint, MYC targets, and DNA repair were strikingly enriched, while immune response-related pathways were relatively inhibited, which was consistent with high tumor proliferation rate of C1. The crosstalk among activated cell cycle pathways, genomic instability and oxidative stress promoted the tumor progression and thus led to unfavorable outcome in C1 subtype. Oncogenic pathways such as WNT, TGF-β, Notch, Hedgehog, angiogenesis, hypoxia, and EMT were more enriched in C2 subtype compared with other subtypes. Lines of evidence have verified the role of these oncogenic pathways in the regulation of TME and response to immunotherapy (Yang et al., 2010; Albini et al., 2018; Meurette and Mehlen, 2018; Patel et al., 2019; Gampala and Yang, 2021). Immune response pathways such as interferon response, IL2-STAT5 signaling, complement, IL6-JAK-STAT3 signaling, and inflammatory response were much enriched in C3 and C4 subtypes, which were responsible for their favorable prognosis. In addition to immune response pathways, metabolic pathways such as fatty acid metabolism, adipogenesis, and bile acid metabolism were also enriched in C4 subtype. The metabolic alterations have been demonstrated to shape TME components thereby influencing tumor progression and immunotherapy efficiency (Lyssiotis and Kimmelman, 2017; DeBerardinis, 2020).

Cancer patients with different TME may have different response to immunotherapy. We estimated the potential response of four subtypes to immunotherapy by using immunotherapy-related indicators (T cell inflamed GEP, Th1/IFN-γ, and cytolytic activity). Four subtypes showed distinct enrichment of these indicators where C3 subtype was predicted to benefit most from immunotherapy. In addition, four subtypes also displayed differential expression of key immune checkpoints such as PD-1, CD274, CTLA-4, and LAG3. C3 subtype exhibited the highest expression of these checkpoints, suggesting C3 subtype was sensitive to ICIs. From the above results, we concluded that the TME-based subtyping was effective to provide a guidance for LUAD patients receiving immunotherapy.

Furthermore, we established a TME-related risk model containing five prognostic genes (PTTG1, MS4A1, ZNF750, RHOV, and KRT6A) for predicting LUAD survival. PTTG1 was found to promote lung cancer migration and invasion (Li et al., 2013), and knockdown of PTTG1 could enhance anti-tumor activity in LUAD (Chen et al., 2021). The expression of MS4A1 was shown to be positively correlated with the survival of colorectal carcinoma (Mudd et al., 2021), which was consistent with our result that MS4A1 was highly expressed in low-risk group. ZNF750 is a tumor suppressor in squamous cell carcinoma, which can suppress cell migration (Hazawa et al., 2017). In our study, ZNF750 expression level was downregulated in high-risk group, which supported its protective role in inhibiting tumor progression. RHOV was shown to facilitate tumor cell growth and metastasis in LUAD (Zhang et al., 2021). In our results, RHOV was evidently elevated in high-risk group. Overexpression of KRT6A was able to promote LUAD cell proliferation through EMT process (Yang et al., 2020), which may lead to poor prognosis in high-risk group. Previous studies have illustrated that the five prognostic genes are involved in tumor progression and migration in lung cancer or other cancer types, implying that our TME-related risk model was reliable to predict LUAD prognosis. ROC curve analysis showed a high AUC and validated the efficiency of the risk model. In addition, we evaluated the predictive value of the risk model in guiding immunotherapy and chemotherapy. Two risk groups showed differential immune responses to immunotherapy and differential IC50 to four chemotherapeutic drugs (docetaxel, vinorelbine, paclitaxel, and cisplatin), which illustrated that the risk model also had a potential in assisting the decision-makings in immunotherapy and chemotherapy.

## Conclusion

In conclusion, our study revealed the molecular characteristics of LUAD patients based on TME-related signatures. The distinct biological pathways and TME features of four TME-based subtypes laid a foundation for the further exploration of the crosstalk among TME, genomic instability, and oncogenic pathways in LUAD. The TME-related risk model was efficient and reliable to predict LUAD prognosis and assist clinical treatment.

## Data Availability

The original contributions presented in the study are included in the article/Supplementary Material, further inquiries can be directed to the corresponding authors.
