# Supplementary figures and images for: Molecular characterization based on tumor microenvironment-related signatures for guiding immunotherapy and therapeutic resistance in lung adenocarcinoma
Source: Front Pharmacol. 2023 Jan 16;14:1099927. doi: 10.3389/fphar.2023.1099927 (PMC9884810; doi:10.3389/fphar.2023.1099927)

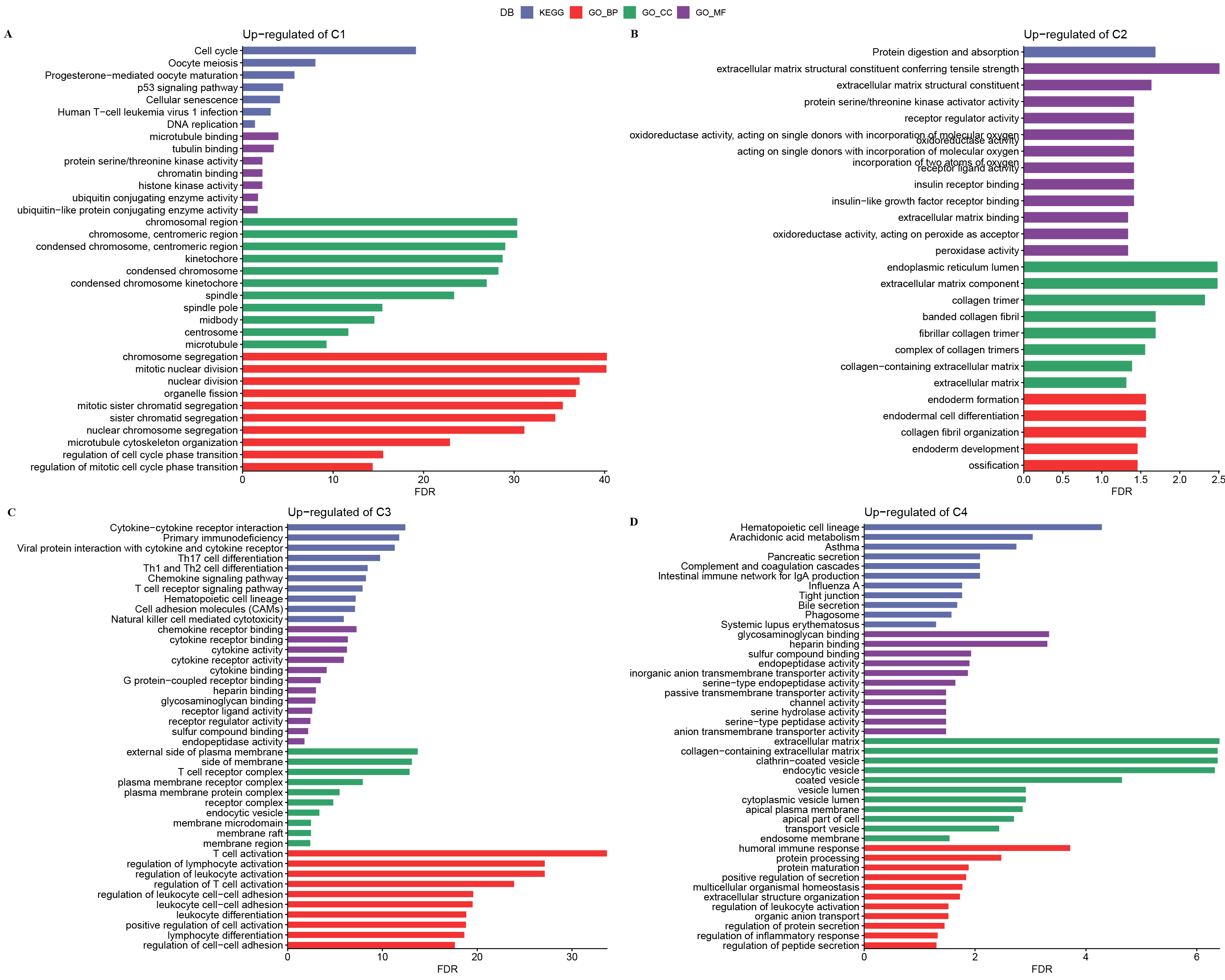

Supplement: Supplementary file 1 [file Image3.JPEG]

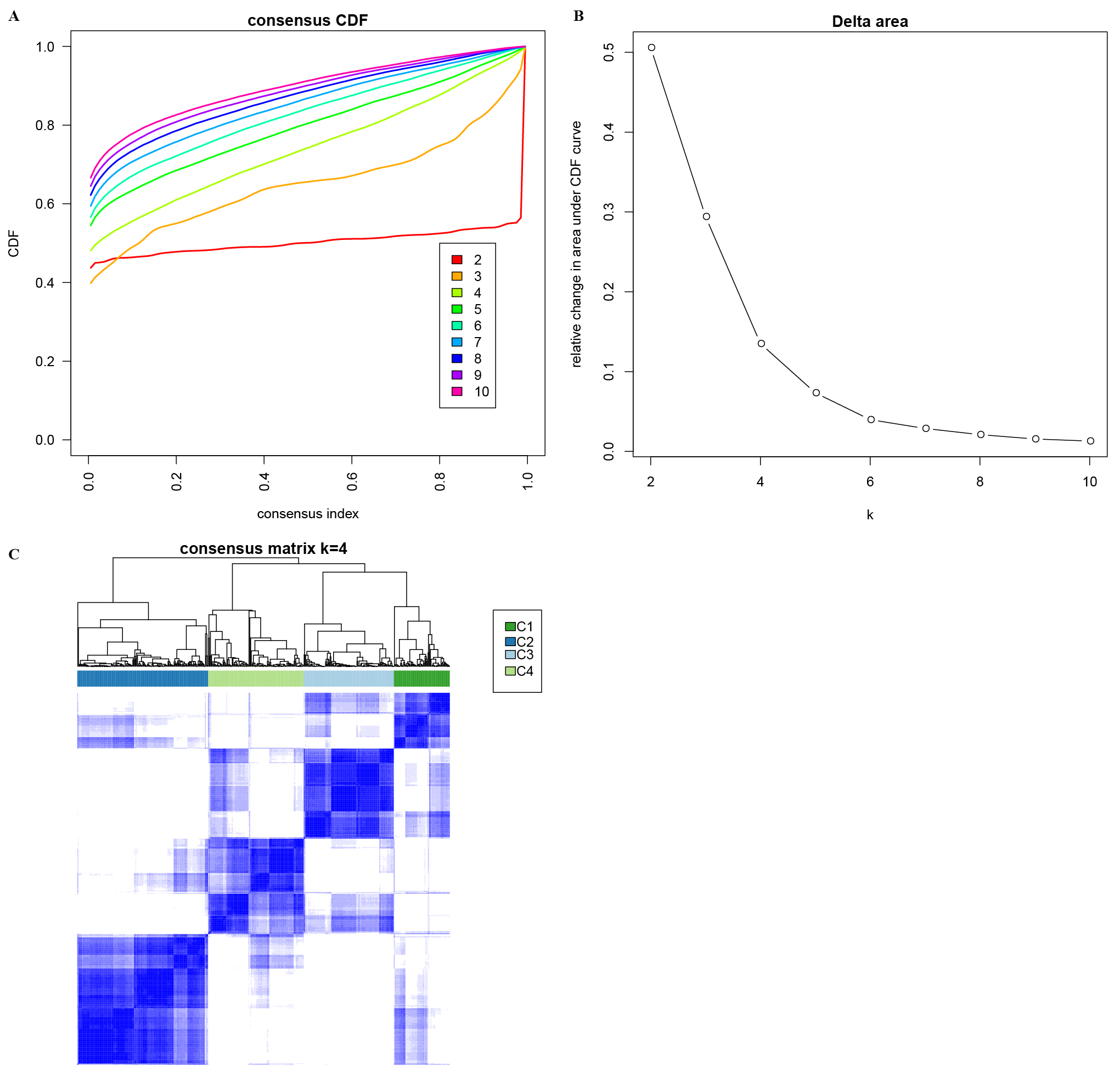

Supplement: Supplementary file 2 [file Image1.JPEG]

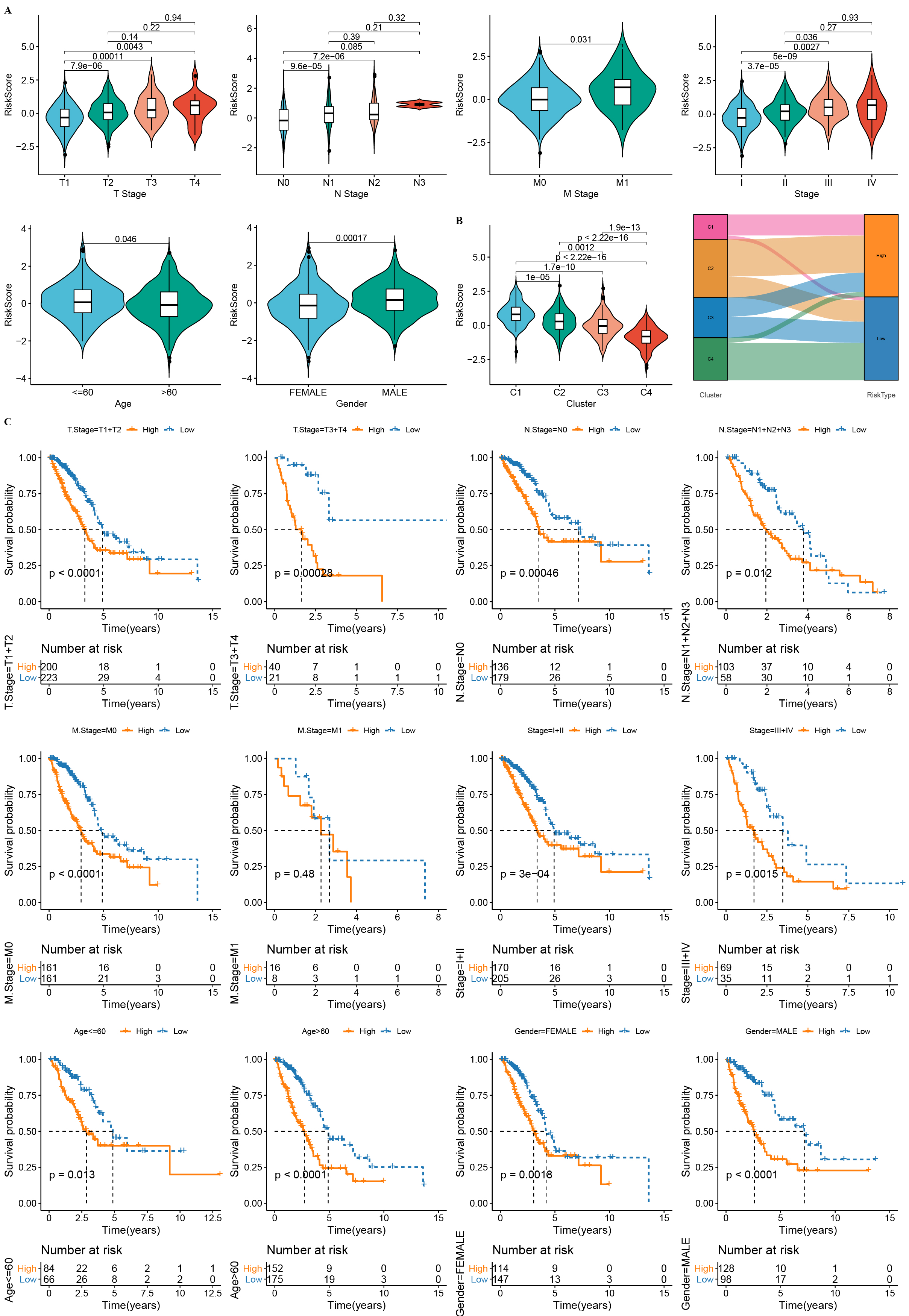

Supplement: Supplementary file 3 [file Image4.JPEG]

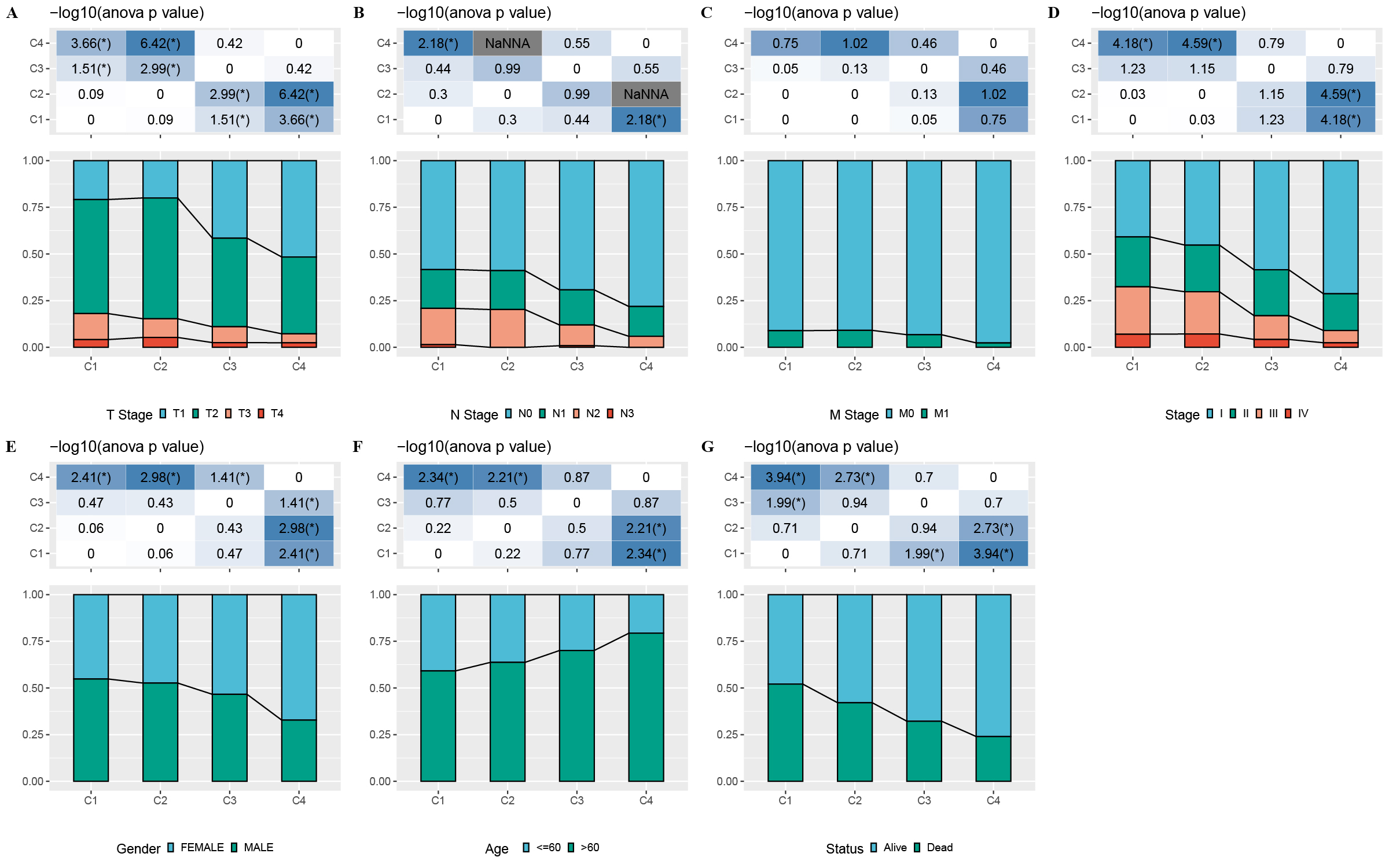

Supplement: Supplementary file 4 [file Image2.JPEG]

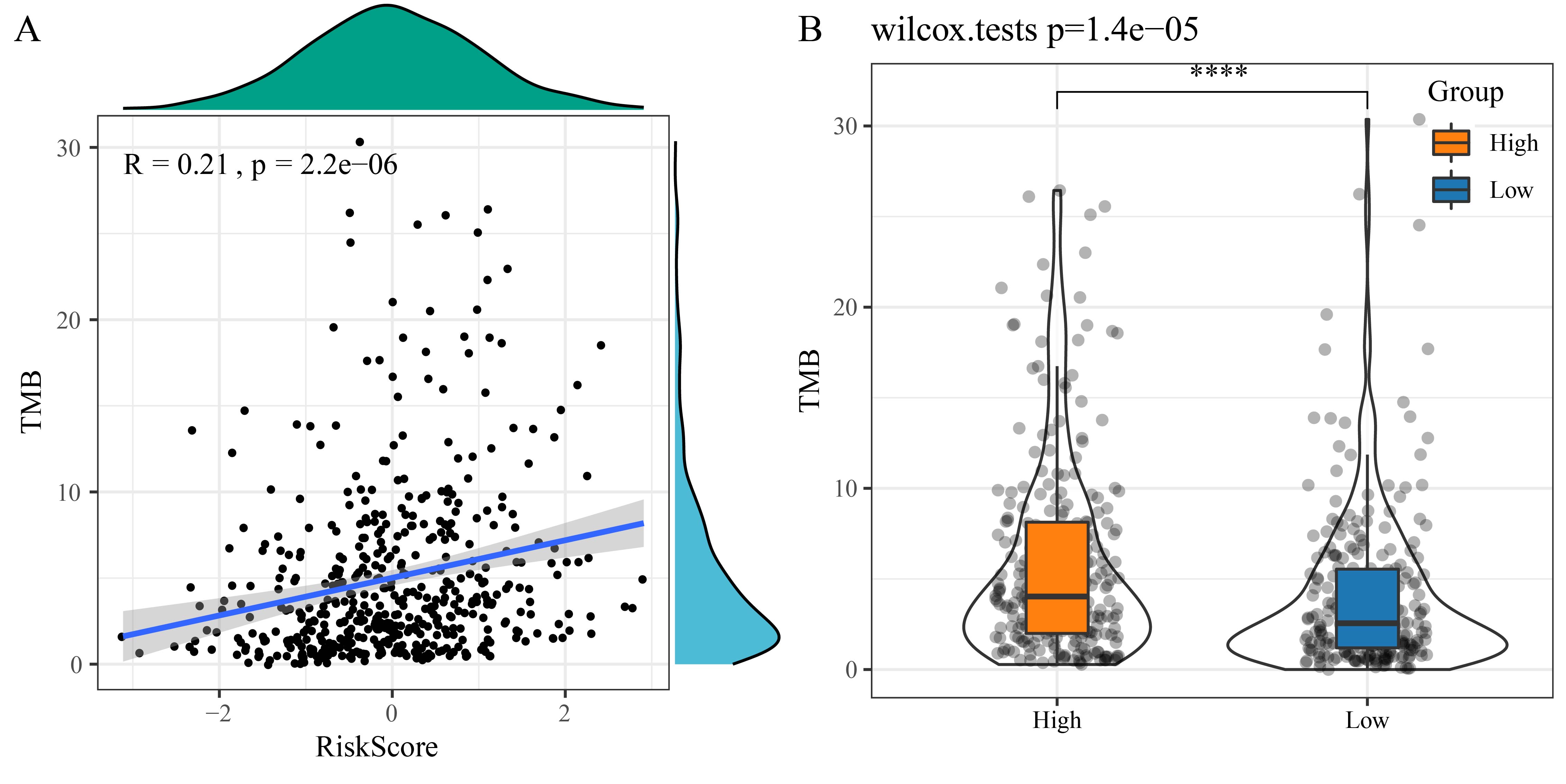

Supplement: Supplementary file 5 [file Image5.JPEG]
