# Supplementary material for: Molecular characterization based on tumor microenvironment-related signatures for guiding immunotherapy and therapeutic resistance in lung adenocarcinoma
Source: Front Pharmacol. 2023 Jan 16;14:1099927. doi: 10.3389/fphar.2023.1099927 (PMC9884810; doi:10.3389/fphar.2023.1099927)
Supplement: Supplementary file 6 [file Table2.DOCX]

For the data analyzed in this study please see:

https://www.jianguoyun.com/p/DXUbvjoQ3I2UCxiFvOYEIAA
