# Supplementary material for: Molecular characterization based on tumor microenvironment-related signatures for guiding immunotherapy and therapeutic resistance in lung adenocarcinoma
Source: Front Pharmacol. 2023 Jan 16;14:1099927. doi: 10.3389/fphar.2023.1099927 (PMC9884810; doi:10.3389/fphar.2023.1099927)
Supplement: Supplementary file 7 [file Table3.DOCX]

Supplementary materials

Figure S1. Identification of four TME-based molecular subtypes in TCGA dataset by unsupervised consensus clustering. (A) CDF curves of different cluster numbers. (B) Relative change in area under CDF curve of different cluster numbers. (C) Consensus matrix when cluster number k = 4.

Figure S2. The distribution of clinical characteristics in four subtypes in TCGA dataset. *P < 0.05.

Figure S3. GO and KEGG enrichment analysis of upregulated genes in four subtypes in TCGA dataset.

Figure S4. The relation of risk score with clinical characteristics. (A) The risk score in different clinical characteristics. (B) The risk score of four subtypes and Sankey plot of risk type and subtypes. (C) Survival plots of high-risk and low-risk groups in different clinical characteristics.

Figure S5. The risk score was positively correlated to TMB and high group had enhanced TMB.

Table S1. A list of 418 prognostic genes from univariate Cox regression analysis.
